# The utility of biomedical scaffolds laden with spheroids in various tissue engineering applications

**DOI:** 10.7150/thno.58421

**Published:** 2021-05-03

**Authors:** SooJung Chae, Jiyoung Hong, Hanjun Hwangbo, GeunHyung Kim

**Affiliations:** 1 Department of Biomechatronic Engineering, College of Biotechnology and Bioengineering, Sungkyunkwan University (SKKU), Suwon 16419, Republic of Korea.; 2 Biomedical Institute for Convergence at SKKU (BICS), Sungkyunkwan University, Suwon 16419, Republic of Korea.

**Keywords:** spheroid, tissue engineering, spheroid application, scaffold-free, scaffolded

## Abstract

A spheroid is a complex, spherical cellular aggregate supporting cell-cell and cell-matrix interactions in an environment that mimics the real-world situation. In terms of tissue engineering, spheroids are important building blocks that replace two-dimensional cell cultures. Spheroids replicate tissue physiological activities. The use of spheroids with/without scaffolds yields structures that engage in desired activities and replicate the complicated geometry of three-dimensional tissues. In this mini-review, we describe conventional and novel methods by which scaffold-free and scaffolded spheroids may be fabricated and discuss their applications in tissue regeneration and future perspectives.

## Introduction

A spheroid is a three-dimensional (3D), self-assembling cell aggregate 1. Before spheroids were introduced by Moscona and Moscona 1, conventional two-dimensional (2D) cell cultures created in Petri dishes or culture flasks were widely used; these were convenient, inexpensive, and very reproducible. All cells received identical nutrients. 2D cell cultures have found many applications in *in vitro* studies, disease modeling, analyses of cellular responses, drug screening, and regenerative medicine 2, 3. However, 2D cultures do not mimic the *in vivo* 3D microenvironment; cell-cell and cell-matrix interactions are limited 4. 2D monocultures also do not engage in* in vivo* cellular behavior 5 and are much more drug-sensitive than are spheroids 6, 7, reflecting differences in physical and physiological properties, the expression and spatial organization of surface receptors, cancer gene expression levels, cell stage, drug accessibility, and the local pH 8. Spheroids thus find many applications in tissue engineering (TE) 9. Spheroids feature extensive cellular interactions, mimicking the *in vivo* extracellular matrix (ECM) that mechanically supports cells and regulates most cellular functions, including migration, survival, proliferation, and differentiation. The ECM interacts with growth factors and facilitates contact between cells and ECM components 10. The geometrical similarity between spheroids and cells of native tissue may allow spheroids to mimic cellular functions (including viability and differentiation) better than do monolayer cultures 11, 12. Furthermore, spheroids exhibit higher-level tissue-specific gene expression compared to monocultures, thus affecting the regulation of cell signaling and cytokine expression, because they more closely mimic *in vivo* microstructures 13. Finally, as spheroids are cell aggregates, they protect cells from unwanted external stimuli or drug inflows by ensuring the maintenance of a dense ECM network. Spheroids more closely resemble the geometrical and physiological *in vivo* microenvironment than do monolayers, enhancing their clinical and regenerative utility.

However, spheroids are associated with critical shortcomings attributable to their geometrical structure. Cells rely exclusively on diffusion to transport nutrients or eliminate waste. Therefore, the interiors of spheroids lack nutrients and oxygen and retain CO_2_, ultimately triggering necrosis, whereas the cells in the outer layer successfully transfer nutrients and remove waste 14, 15. Necrosis usually develops in spheroids of larger diameters, e.g., 400 to 600 µm, and reduces cell viability and proliferation 16. To overcome these limitations, efforts have been made to artificially supply oxygen via fiber injection 17-20. Moreover, spheroids scatter light and feature inhomogeneous dye diffusion 21, rendering imaging very challenging. In efforts to counter these limitations, cryoplastic sectioning, light-sheet fluorescence microscopy, and optical clearing methods have been employed 21, 22.

Furthermore, it is difficult to accurately shape spheroids and obtain complex 3D cell constructs. Nonetheless, spheroids have been widely used for the regeneration of skin, blood vessels, nerves, and liver tissues. Some tissues (such as cardiac tissues) require extensive cell-ECM interactions to maximize tissue-specific functions 23. Spheroids have thus been incorporated into various biomedical scaffolds (made of collagen, elastin, or ECM), yielding complex 3D structures. However, the scaffold materials may trigger immune responses and cause infections 24-27.

Several previous works have shown that spheroids can be used for various TE applications, alone or with a biomedical scaffold fabricated using conventional or novel processes such as electrospinning, 3D printing, and hydrogel contraction. In this mini-review, we explore the use of spheroids for the regeneration of bone, cartilage, liver tissues, nerves, and skin, dealing separately with scaffold-free spheroids and scaffolded spheroids. We describe their advantages and disadvantages and summarize future research directions.

## Spheroid Fabrication Methods

Spheroid formation does not require a special culture medium and can be divided into three steps: loose cellular aggregation caused by interactions between integrin and the ECM, accumulation of cadherin, and the formation of compact spheroids via cadherin-cadherin interactions 28. Cell adhesion molecules such as cadherins and integrins play significant roles in spheroid formation 29. During the first stage, the ECM binds single cells via their membrane integrins, and the cells become loosely aggregated. E-cadherin, the principal glycoprotein of Ca^2+^-dependent systems, mediates homotypic cell-cell adhesion 30 and triggers robust spheroid compaction after cell accumulation. Spheroids can be fabricated by various methods, using the same culture media as those of 2D culture. We next briefly review conventional spheroid fabrication techniques such as the hanging drop, nonadherent surface, spinning flask, and rotating vessel methods, as well as the more recently developed microfluidic, acoustic, water-in-water emulsion, and 3D printing methods.

**Figure 1:**
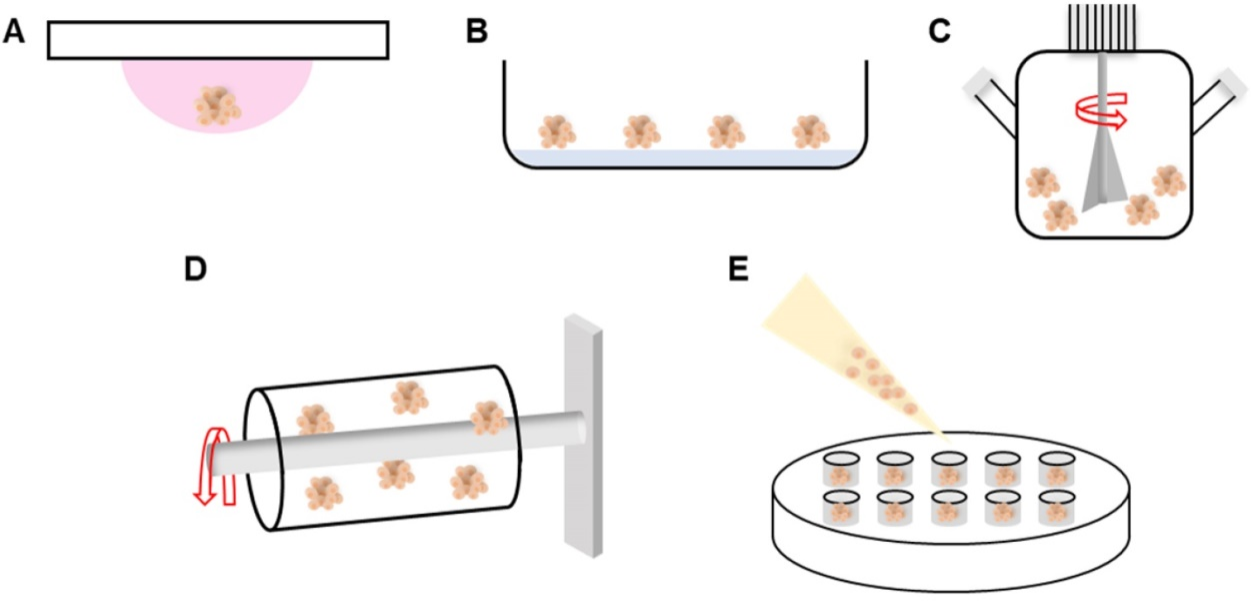
**Schematic of various spheroid fabrication methods**.** A.** Hanging drop. **B.** Nonadherent surface. **C.** Spinning flask. **D.** Rotating vessel. **E.** Micro-patterned mold.

The hanging drop, nonadherent surface, spinning flask, rotating vessel, external force-assisted, and matrix-embedded methods have all been employed to induce cell aggregation. The hanging drop method is cost-effective; cells aggregate within suspended droplets under the influence of surface tension and gravitational forces (**Figure 1A**) 31, 32. The spheroid size is determined by the droplet volume. However, the procedure is labor-intensive and thus unsuitable for high-throughput production. With the nonadherent surface method, spheroids are readily fabricated in an environment in which cells cannot adhere. Although no special equipment is required, the process is inefficient and spheroid size cannot be controlled (**Figure 1B**) 33. In the spinning flask method, cells collide within rotating bioreactors, triggering aggregation (**Figure 1C**). The centripetal force promotes nutrient transfer and waste removal, but the high shear stress can damage or kill cells 34, 35. To minimize shear stress, the rotating vessel maintains the cells in continuous suspension (**Figure 1D**) 36, 37. Recent fabrication methods have employed micro-patterned molds (**Figure 1E**) 38. Spheroids are fabricated by seeding cells onto a non-adhesive mold material. However, the spheroid size is variable, throughput is low, and handling is difficult 39. To overcome these limitations, methods such as microfluidics, micromolded non-adhesive hydrogels, acoustic, and water-in-water emulsions methods have been implemented to fabricate spheroids.

**Figure 2:**
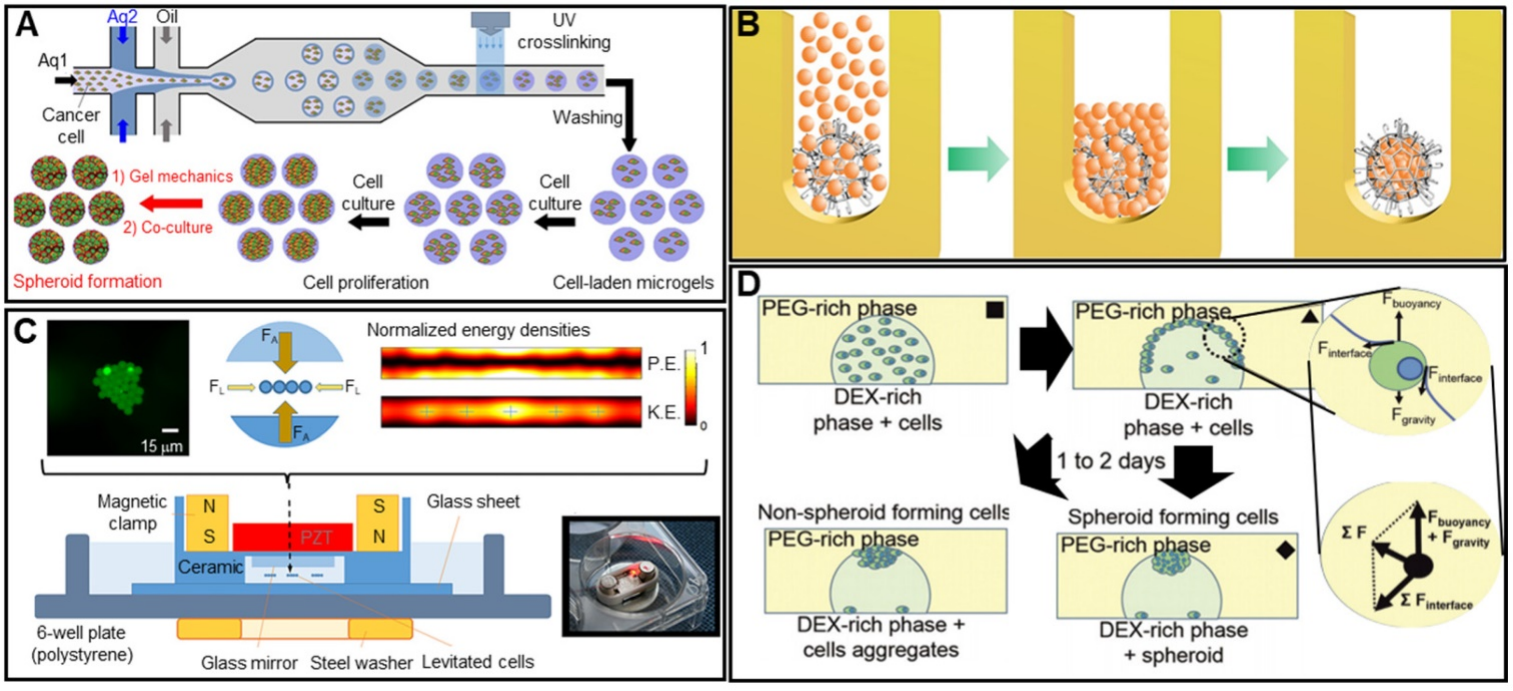
** Spheroid fabrication methods. A.** Schematic images of microfluidic device (top) and optical images of the fabricated spheroids (bottom). Adapted with permission from 42, copyright 2018 MDPI. **B.** Schematic illustration of the spheroid fabrication process using micromolded non-adhesive hydrogel. Adapted with permission from 47, copyright 2016 Public Library of Science. **C.** Schematic of cell aggregation caused by acoustic stimulation. Adapted with permission from 50, copyright 219 Springer Nature. **D.** Schematic illustration of water-in-water emulsion method using phase separation between PEO and dextran. Adapted with permission from 52, copyright 2020 Royal Society of Chemistry.

In microfluidics, low fluid volumes are processed, allowing multiplexing, automation, and high-throughput screening. Emulsions, microwells, and U-shaped microstructures have been used to fabricate spheroids (**Figure 2A**) 40-42. Spheroid fabrication features cell encapsulation, with two fluids forming droplets and cells encapsulated in a hydrogel 43, 44. Micromolded non-adhesive hydrogels are used in combination with the hanging drop, rotating vessel, and microfabrication techniques; spheroids are fabricated as desired by seeding cells into micromold chambers formed from non-cell-adhesive polymers such as a polyacrylamide hydrogel or an agarose gel (**Figure 2B**) 45-47. A microfluidic system can be supplemented with an acoustic wave generator, such that injected cells aggregate in the trough of a wave (**Figure 2C**). This allows high-throughput production, but the equipment is expensive 48-50. In the water-in-water emulsion method, cells are injected into two different phases of an aqueous solution; an osmotic effect then triggers aggregation (**Figure 2D**). This method is cost-efficient and enables high-throughput production 51, 52. More recently, 3D printing technology has emerged as a novel fabrication strategy; this is reproducible, allows high-throughput production, and is precise. In particular, a drop-on-demand method has been employed to fabricate a non-adhesive hydrogel cup. In this process, a hydrogel bio-ink is deposited layer-by-layer and cell droplets are placed in the center of the hydrogel cup. Over time, the cells aggregate into spheroids 53. The precise spheroid fabrication capacity of 3D printing has greatly aided spheroid research. Kang et al. reported precise positioning of HepG2 and NIH3T3 spheroids (at the micrometer scale) using a 3D bio-dot printing method 54. Such spheroids can be used alone for TE or combined with biocompatible supporting materials that remedy spheroid shortcomings or provide additional functions. In this review, we named the former as “scaffold-free” and the latter as “scaffolded”.

## Spheroid applications in TE

TE seeks to develop implantable biological substitutes that maintain or improve tissue function 55. Many researchers have shown that mimicking the physiological environment of native tissue yields superior results. Therefore, spheroids have been increasingly used for TE, as their 3D form mimics native tissue. **Scaffold-free** approaches use spheroids alone, whereas **scaffolded** approaches feature scaffolds. Both approaches have unique advantages and disadvantages, but researchers have been versatile, as shown below.

### Scaffold-free applications

Cell sheets, spheroids, or tissue strands are implanted alone (**Table 1**) 56. The spheroids serve as building blocks, with spheroids fusing to form larger 3D structures. The high initial cell density enables spheroid self-assembly. Immune responses to scaffold by-products are obviously absent. However, the absence of supporting material can create significant mechanical problems in dynamic *in vivo* applications. Scaffold-free strategies are commonly used for tissue self-assembly and microchannel fabrication 57-60. In the former approach, spheroids are placed in a mold/support to obtain the desired macrostructure. The latter approach is typically used for drug testing or to observe cellular activities such as vascularization.

#### Bone

**Figure 3:**
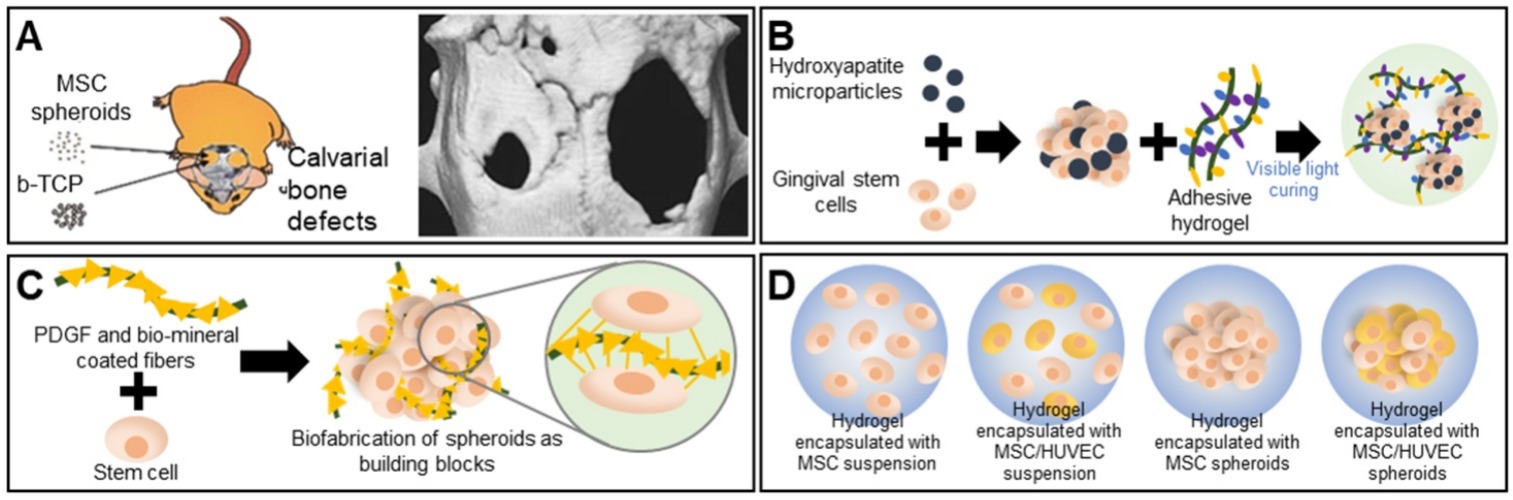
** Scaffold-free spheroid applications for bone regeneration. A.** The experimental concept (left) and micro-CT images of a rat skull at 8 weeks after spheroid implantation (right). Adapted with permission from 65, copyright 2015 Springer Nature. **B.** Schematic overview of the aggregation of gingival stem cells, HA, and an adhesive hydrogel 66. **C.** Schematic illustration and osteogenic differentiation of a spheroid building block including biominerals and PDGF-coated PLLA fibers 68. **D.** Schematic illustration of spheroid encapsulation within a collagen/fibrin matrix 69.

Bones support weight and protect organs; bones are subjected to continuous mechanical stresses that can trigger fractures or osteoporosis 61, 62, particularly in the elderly, who thus require bone regeneration. The cell type, biomaterial, bone architecture, and mechanical properties must all be considered 63. Calcium phosphate-based ceramics such as hydroxyapatite (HA) and beta-tricalcium phosphate (β-TCP) are widely used as scaffolds because they are osteoconductive 64. Ceramics are commonly combined with spheroids to enhance osteogenic differentiation. Suenaga et al. investigated the effects of β-TCP on mesenchymal stem cell (MSC) spheroids used for *in vivo* calvarial regeneration, with the test components comprising MSC spheroids, β-TCP, and an MSC-spheroid/β-TCP composite. Surprisingly, the MSC spheroids afforded better bone regeneration than did the composite. The persistence of β-TCP particles between newly formed bone tissues hindered uniform bone formation. The computed tomography (CT) image in **Figure 3A** displays the bone regeneration of calvarial defects implanted with MSC spheroids (left) versus that of untreated defects (right) 65. A recent report found that the addition of nano-hydroxyapatite particles (nHAps) to spheroids enhanced bone regeneration. Hasani-Sadrabadi et al. prepared an adhesive hydrogel (AdhHG) with tunable mechanical properties by modifying methacrylated alginate with dopamine and adding an arginine-glycine-aspartate (RGD) motif (**Figure 3B**). To induce osteogenesis, nHAps were incorporated into MSC spheroids encapsulated in AdhHG. Spheroids of MSC cells aggregated with HA and AdhHG to afford significantly improved tissue mineralization and bone-filling at 8 weeks after implantation, compared to the alginate hydrogel, which was attributable to the enhanced crosslinking capacity of the modified AdhHG. The resultant mechanical properties were better 66.

Bone TE requires adequate nutrient transfer through vascularized connective tissue 67. Osteogenic expression improved when human adipose-derived stem cell (ASC) spheroids and poly(L-lactic acid) (PLLA) fibers were coated with platelet-derived growth factors (PDGFs) and biominerals (**Figure 3C**) 68. The synergistic actions of the angiogenic growth factors and biominerals enhanced vascularized bone regeneration. Heo et al. used MSC/human umbilical vein endothelial cell (HUVEC) spheroids as building blocks for bone TE. The MSC/HUVEC spheroids were encapsulated in a collagen/fibrin hydrogel. Cell viability, proliferation, and osteogenic differentiation were enhanced compared to the three control groups consisting of hydrogels encapsulated with an MSC suspension, an MSC/HUVEC suspension, and MSC spheroids (**Figure 3D**) 69.

#### Cartilage

**Figure 4:**
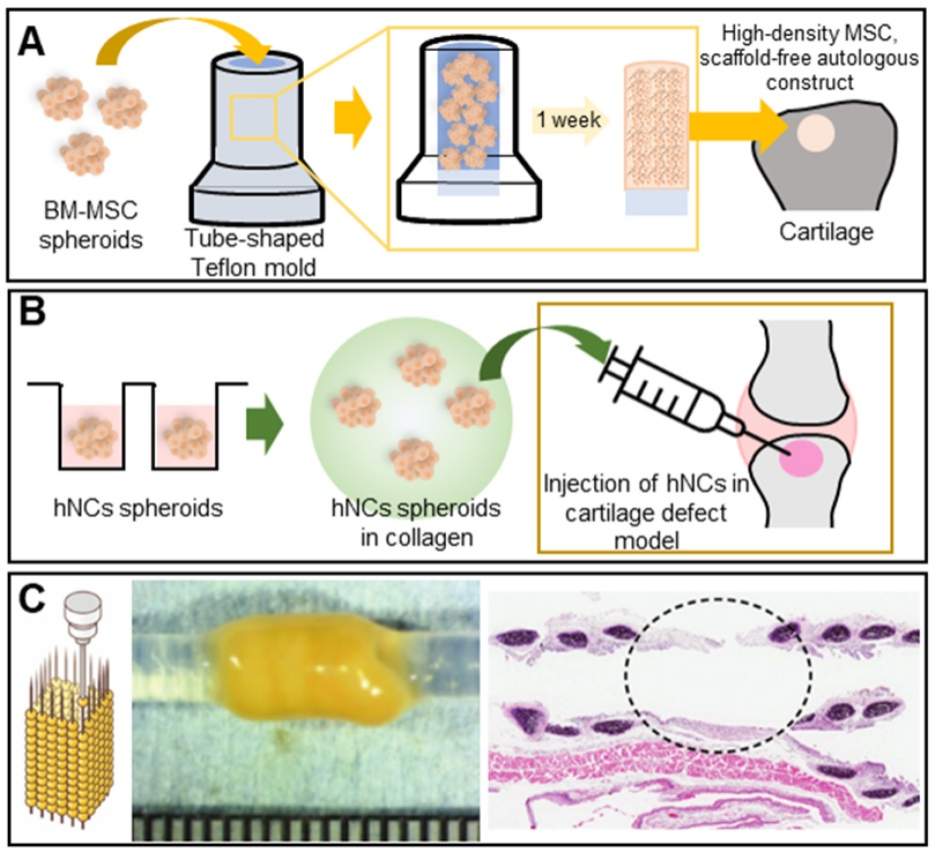
** Scaffold-free spheroid applications for cartilage regeneration. A.** Schematic illustrations of a cylindrical construct composed of a fused spheroid and implantation 73. **B.** Schematic of MSC spheroid injection with collagen in a cartilage defect model 74. **C.** Illustration (left) and an optical image of cartilaginous tubes fabricated using the Kenzan method (middle), and an H&E-stained image of the graft at 35 days after transplantation (right). Adapted with permission from 78, copyright 2019 John Wiley and Sons.

Cartilage is a protective, blood vessel-free connective tissue located between bones or joints that softens impacts and supports bony motion. The self-repair capacity is limited but cartilage is not easily damaged 70, 71. However, constant loading or stress may cause wear, and a decrease in glucosamine synthesis with aging may reduce the levels of proteoglycans, the principal components of articular cartilage, triggering degenerative disease 72. Several studies have shown that spheroids better enhance cartilage regeneration than do 2D cultured cells. Ishihara et al. used bone-marrow-derived MSC (BM-MSC) spheroids to regenerate osteochondral defects in rabbit knee joints. The spheroids were loaded into tube-shaped Teflon molds to form cylinders. *In vivo,* the spheroids differentiated into both bone and cartilage (**Figure 4A**) 73. Human nasal septum-derived chondrocyte (hNC) spheroids repaired osteochondral defects both *in vitro* and *in vivo*, resulting in improved cell viability, proliferation, and gene expression, as well as generated more ECM components, compared to a 2D monoculture of hNCs (**Figure 4B**) 74.

The cartilaginous trachea sends air to the lungs; thus, tracheal defects are serious 75, 76. Tracheal TE is considered ideal in terms of tracheal replacement 77. Machino et al. constructed a trachea-like tube composed of various cell types 78. Artificial tracheae featuring two types of hybrid spheroids - one consisting of fibroblasts, HUVECs, and MSCs, and the other consisting of chondrocytes, HUVECs, and MSCs - were transplanted into rats. Immunohistological staining revealed the development of an epithelium and capillary network similar to those of the native trachea (**Figure 4C**). Complete tubular grafting of human cells may become possible.

#### Liver

**Figure 5:**
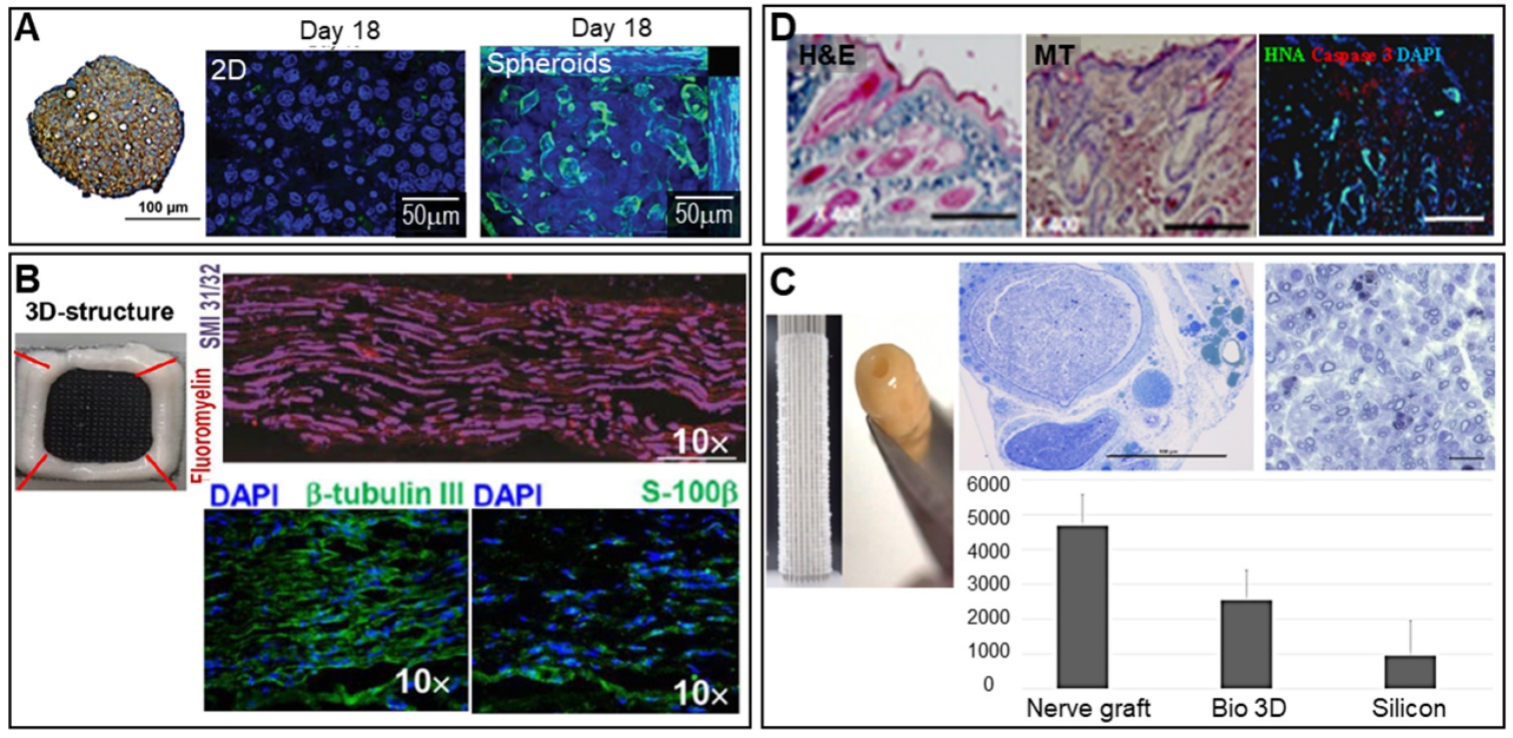
** Scaffold-free spheroid applications for liver, nerve, and skin regeneration. A.** Spheroids stained for CPS1 [a periportal marker (brown)] and with hematoxylin (blue) (left). An MRP-2 (green)/Hoechst (blue) immunofluorescence image of 750 C3A cells in 2D culture (middle) and a spheroid observed after 18 days of culture (right). Adapted with permission from 80, copyright 2016 Oxford University Press. **B.** Optical image of 3D-bioprinted scaffold-free nerve constructs obtained from GMSC spheroids using a needle array (top left), and SMI-31/32/fluoromyelin- (top right), DAPI/β-tubulin III- (bottom left), and DAPI/S-100β-immunostained images (bottom right) (scale bars: 200 mm). Adapted with permission from 58, copyright 2018 Springer Nature. **C.** Macroscopic image of spheroids robotically inserted into the needle array (top left) and the constructs obtained after spheroid fusion, with the histology of regenerated nerves visualized using toluidine blue (top middle and right) and the number of axons observed in each group (bottom) (scale bar: 1 mm). Adapted with permission from 85, copyright 2019 John Wiley and Sons. **D.** DAPI-, caspase-3-, HNA -immunostained images obtained on day 14 (right) (scale bar: 100 mm), and H&E- (left) and Masson's trichrome-stained (middle) images of the wound bed on day 14 (scale bars: 500 mm). Adapted with permission from 89, copyright 2015 Public Library of Science.

The liver performs vital regulatory functions; produces bile acids; engages in hormone excretion, fat and protein metabolism, and vitamin and mineral storage; and exerts other physiological functions 79. Liver cell spheroids are commonly used for drug testing. C3A hepatocarcinoma cell spheroids were fabricated using an overlay method 80 and exhibited *in vivo*-like features such as 3D cell-cell interactions, zonation, and structural and functional polarization. Moreover, the spheroids expressed and secreted significantly higher levels of albumin and urea compared to a 2D monoculture (**Figure 5A**).

Liver fibrogenesis is caused by injury and characterized by the excessive deposition of ECM 81. The differentiation of hepatic stellate cells (HSCs) into collagen-secreting myofibroblasts activates fibrosis, as does hepatocyte (HEP) damage. HSC/HEP cell spheroids are used to test drugs that may counter fibrosis, as the spheroids exhibit *in vivo*-like HSC behavior 82.

#### Nerves

The brain is composed of neurons, glial cells such as astrocytes, microglia, oligodendrocytes, pericytes, and endothelial cells. Brain disease often develops after a brain or spinal cord injury, or may be neurodegenerative in nature (Parkinson's, Alzheimer's, or Huntington's disease) 83, 84. Spheroids may be used to effectively deal with these conditions. For example, as shown in **Figure 5B**, human gingiva-derived MSC (GMSC) spheroids stacked in a needle array and immunostained for axonal SMI-31/32 (neurofilament H) markers produced signals indicative of possible peripheral nerve regeneration 58. An autologous, 3D nerve conduit graft consisting of human normal dermal fibroblast (hNDFB) spheroids regenerated peripheral nerve defects 85. The histological analysis results shown in **Figure 5C** show that the myelinated axon diameter and myelin thickness were significantly greater than those of the control (with a silicon graft).

#### Skin

The skin is the largest organ of the human body and is responsible for thermoregulation and protection against solar radiation. However, the skin is susceptible to damage, being the first line of defense against external insult. Natural healing features sequential hemostasis, inflammation, cell proliferation, and cell maturation 86, 87. Umbilical cord tissue-derived MSC spheroids have been used to accelerate this process 88.The immunostaining results of extracellular components (laminin, collagen, and fibronectin) indicated that ECM components were synthesized. The levels of hepatocyte growth factor (HGF), transforming growth factor-β1, granulocyte-colony stimulating factor, vascular endothelial growth factor (VEGF)-A, fibroblast growth factor (FGF)-2, and interleukin-6 were significantly higher than those of a 2D monoculture. Low-level light irradiation induced secretion of angiogenic growth factors (VEGF and FGF) by adipose-derived stromal cell spheroids (**Figure 5D**) 89, 90.

#### Vessels

**Figure 6:**
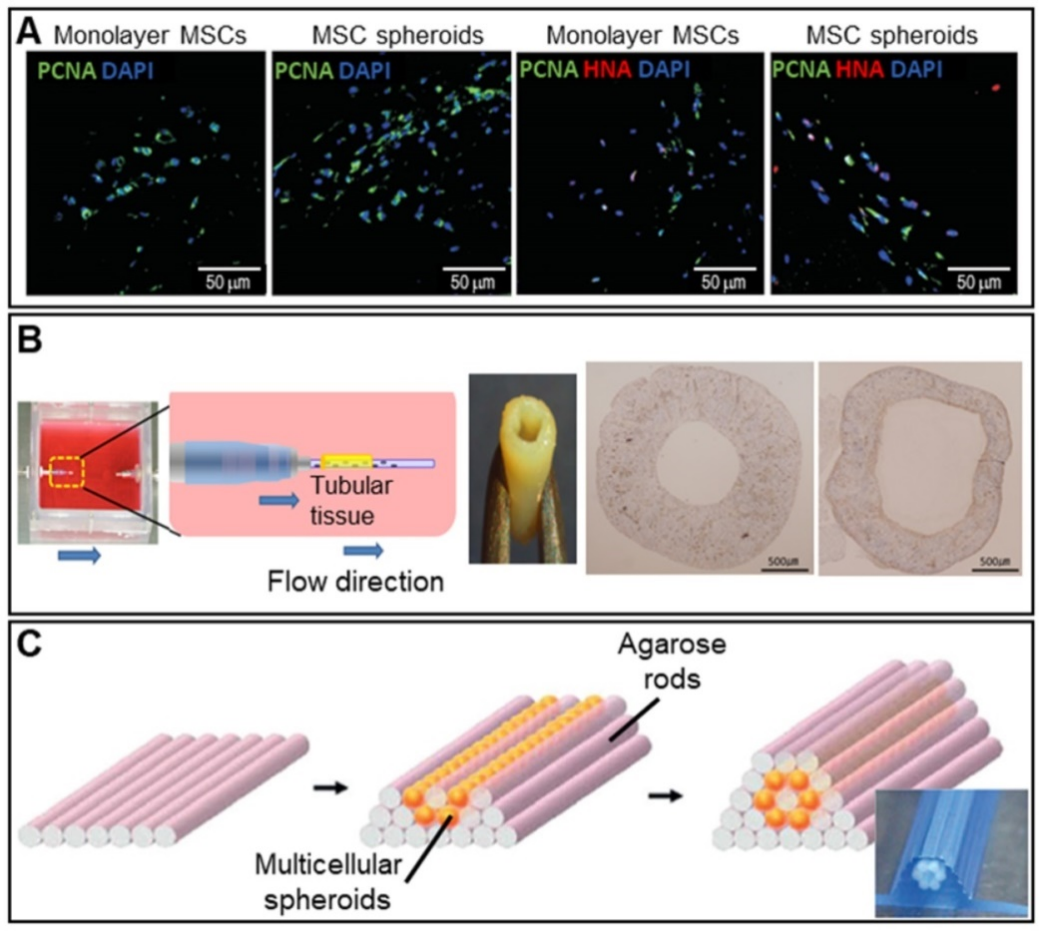
** Scaffold-free spheroid applications for vessel regeneration. A.** DAPI/PCNA and DAPI/PCNA/HNA immunofluorescence-stained images at 3 days after transplantation of monolayer MSCs or MSC spheroids cultured for 7 days (scale bar: 50 mm). Adapted with permission from 93, copyright 2016 Korean Society of Lipidology and Atherosclerosis. **B.** Schematic overview of the bioreactor system (left) and an optical image of the fabricated vascular graft (middle). Von Willebrand factor-stained images taken pre- and post-implantation (right). Adapted with permission from 94, copyright 2015 Public Library of Science. **C.** Schematic illustrations of the tube fabrication composed of multicellular spheroids and agarose rods. Adapted with permission from 95, copyright 2013 John Wiley and Sons.

Angiogenic growth factors including VEGF and stromal cell-derived factors activate various signaling pathways 91, 92. Hypoxia (tissue oxygen deprivation) may develop in the spheroid core; the cells then secrete angiogenic growth factors. **Figure 6A** shows immunofluorescence images of MSC spheroids and a 2D monoculture after hypoxia induction. Proliferating cell nuclear antigen (PCNA) and human nuclear antigen (HNA) markers (cell proliferation markers) were more highly expressed by the spheroids 93.

Many attempts have been made to fabricate tubular structures using spheroids. A 3D bioprinter and skewered spheroids were employed to fabricate 3D tubular structures consisting of HUVECs, human aortic smooth muscle cells, and hNDFBs 94. As shown in **Figure 6B**, the fabricated tubular grafts were then cultured in a dynamic environment featuring perfusion by an endothelial cell medium, a smooth muscle cell medium, and a fibroblast medium, which enlarged the lumen. Norotte et al. used an agarose mold to fabricate a microtubular vascular graft consisting of hybrid spheroids (smooth muscle cells and fibroblasts) (**Figure 6C**) 95. A conventional 3D printer facilitated rapid and reproducible construction of vessels similar to native vessels in terms of both composition and architecture.

#### Other tissues

Scaffold-free spheroids enhance the cell-ECM interactions of cardiac tissues. Scaffold-free cardiac patches consisting of multiple cellular spheroids (human esophageal microvascular endothelial cells, rat neonatal ventricular cardiomyocytes, and hNDFBs) have yielded promising results 96. Cardiac patches grafted into the hearts of nude rats formed vascular networks. However, the contractions were not synchronized to those of native tissue, and some spheroids were inappropriately positioned. To ensure precise positioning, 3D printers have become widely used. A recent vacuum system featuring a bioprinting pipette was used to relocate spheroids of induced, pluripotent stem cell-derived cardiomyocytes in a self-healing support hydrogel to desired locations 97. After removal of the 3D microtissues from the hydrogel, the structure was maintained. Thus, cardiac regeneration may be possible; this is a novel approach toward accurate positioning of individual spheroids. Cardiac spheroids may be used to treat myocardial infarction. Injection of human cardiospheres into the infarct border zones of mice enhanced angiogenesis, tissue protection, and cardiomyocyte proliferation compared to a control group 98.

Hypoparathyroidism is associated with reduced production of parathyroid hormone (PTH), resulting in an imbalance between calcium and phosphorus. Park et al. developed scaffold-free spheroids of differentiated tonsil-derived MSCs (dTMSCs) 99 and implanted them in parathyroidectomized rats expressing a parathyroid-specific protein marker. Spheroid cell viability was reasonable (more than 50%), and high levels of intact PTH were synthesized. Such scaffold-free dTMSCs spheroids are novel and may find clinical application.

Oral ulcers caused by cancer therapies are becoming an increasing concern and may even trigger treatment discontinuation. Ulcer treatment using a 3D ASC spheroid sheet has been investigated 100. An *in vivo* study using white rabbits showed that such a sheet formed a 3D multilayered epithelium that exhibited immunofluorescent staining for CK5 and CK13 (markers of basal and suprabasal cells, respectively), affording better epithelialization and faster wound closure compared to a 2D culture. Thus, the ASC spheroid sheet may effectively treat oral ulcers.

As populations age, periodontitis causing tooth loss has increased 101. Spheroids consisting of a mixture of human periodontal ligament MSCs and HUVECs may reduce periodontitis 102. Spheroid transplantation into rat periodontal tissue defects shows increased bone and cementum formation to a greater extent than did spheroids with only one cell type. Periodontal regeneration may thus be possible.

According to the World Health Organization, lung disease is one of the top five causes of death. Autologous tissue implants may enhance lung regeneration. Recently, Cheng et al. showed that lung spheroids composed of a mixture of progenitor cells and supporting stromal cells from healthy lungs improved pulmonary fibrosis in rats 103. Compared to MSC spheroids, the lung spheroids better reduced fibrotic thickening and tissue infiltration. The same team treated idiopathic pulmonary fibrosis via inhalation of a lung spheroid cell secretome (LSC-Sec) and exosomes. LSC-Sec inhalation reversed the fibrosis caused by bleomycin or silica particles. Currently, idiopathic pulmonary fibrosis remains incurable; hence, the novel inhalation strategy may be very valuable 104.

### Scaffolded Spheroid Applications

A fundamental advantage of scaffolding is the mechanical reinforcement thus afforded to the cells. A scaffold may be formed using tissue-specific ECM and then biomimetically functionalized 56. As shown in **Table 2**, spheroids may be directly seeded into scaffolds or embedded between scaffold layers.

#### Bone

**Figure 7:**
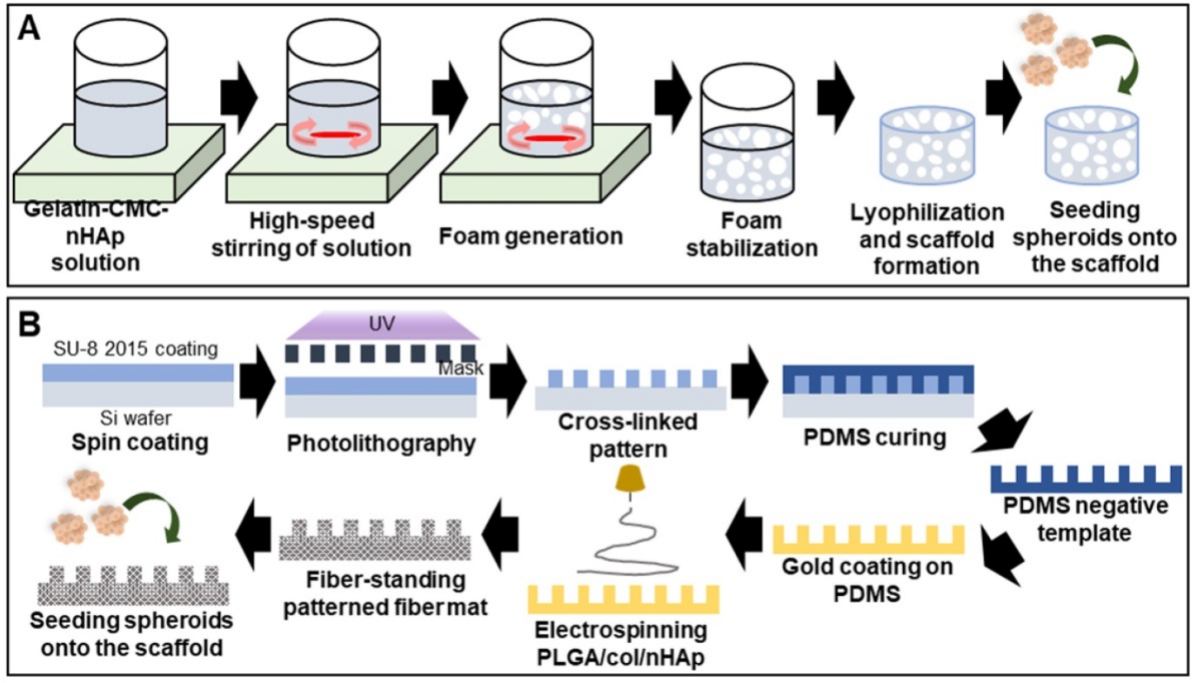
** Scaffolded spheroid applications for bone tissue regeneration. A.** Schematic illustration of macroporous scaffold preparation and macroscopic image of a porous scaffold 106. **B.** Schematic overview of the preparation of free-standing, patterned, electrospun, PLGA/ collagen/nHAp fiber mats 107.

A high scaffold macroporosity (similar to that of native bone) facilitates cellular infiltration and migration and thus, bone regeneration 105. Foaming and freeze-drying techniques are regularly used to ensure macroporosity. A gelatin/carboxymethyl chitosan/nHAp matrix was vigorously mixed to form bubbles and then lyophilized (the SGC scaffold) (**Figure 7A**) 106. The macropore diameter was 500-800 µm, aiding spheroid penetration. Alizarin red S staining indicated enhanced calcium deposition inside the scaffold.

Efficient bone regeneration requires topological scaffold cues. For example, a negatively charged polydimethylsiloxane (PDMS) template was used to fabricate patterned nanofibrous poly(L-glutamic acid) (PLGA)/collagen/nHAp fibers that enhanced osteogenic differentiation (**Figure 7B**) 107. Alkaline phosphatase staining of MSC spheroids seeded into the scaffold was significantly stronger than that of a 2D culture and spheroids cultured on an unpatterned scaffold. The synergistic effects of the patterned scaffold and 3D spheroids, and the fact that the biophysical environment closely resembled that of native tissue, were beneficial.

#### Cartilage

**Figure 8:**
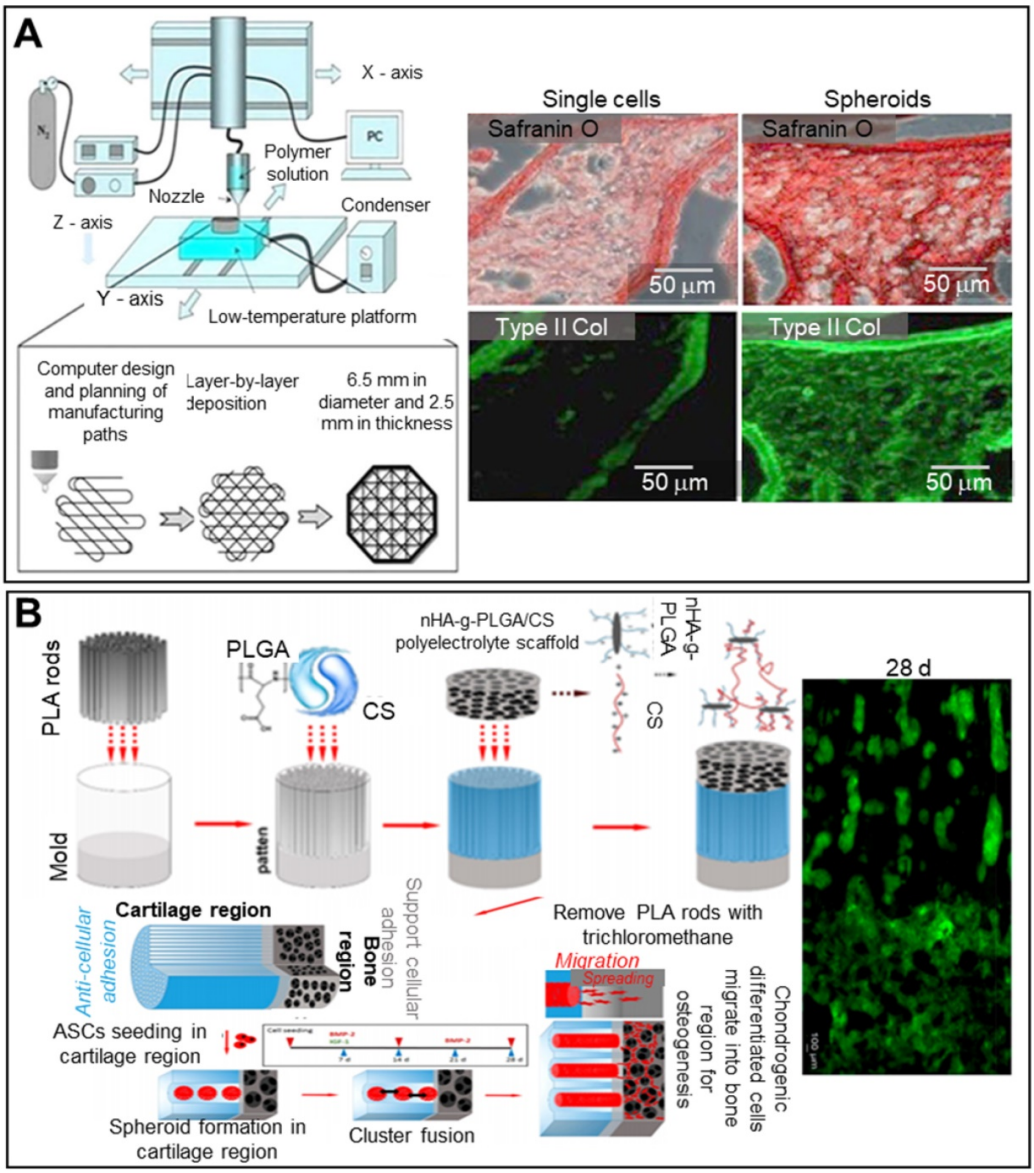
** Scaffolded spheroid applications for cartilage tissue regeneration. A.** Schematic overview of the fabrication of solid freeform scaffolds via frozen liquid deposition (left), with safranin O-stained (red), and type II collagen-stained (green) images, and prelabeled MSC fluorescence images of structures implanted in NOD/SCID mice for 4 weeks (right). Adapted with permission from 110, copyright 2013 AO Research Institute. **B.** Schematic illustration of the fabrication of a bilayer scaffold, and a confocal laser scanning microscopy image of cell distribution in the scaffold at 28 days (right). Adapted with permission from 112, copyright 2020 American Chemical Society.

Subchondral bone regeneration is challenging given the limited self-repair capacity of articular cartilage. To overcome this issue, bi-compartmented (multilayered) therapeutics have been used, with bone morphogenetic protein-2 and MSC spheroids compartmentalized and inserted into sheep osteochondral defects 108. The volume of articular cartilage thus created in the therapeutic group was three-fold that of the untreated group. Maintenance of spheroid morphology is important in terms of effective tissue regeneration 109. Huang et al. thus developed chitosan (CS)-coated, plasma-treated 3D PLGA scaffolds (**Figure 8A**) 110. The scaffolds were seeded with placenta-derived MSC spheroids and transplanted into chondral defects in rabbit knees. Safranin O and collagen II staining (**Figure 8A**) indicated significantly enhanced glycosaminoglycan and type II collagen production in the spheroid group compared to the single-cell group. The scaffold macroporosity preserved the spheroid morphology. PLGA and CS form non-fouling fibrous networks because they are oppositely charged protein-repulsing polyelectrolytes 111. The results shows that the lyophilized scaffold did not re-assume the original hydrogel structure when soaked, thus retaining the porous network. At 14 days after ASC seeding, cells aggregated inside the pores, forming a spheroid. *In vivo* tests (using cartilage defects in rabbit knees) revealed hyaline cartilage of appropriate structure and function, suggestive of cartilage regeneration.

Articular cartilage is a smooth white tissue that forms joints at the ends of bones. To mimic the transition from cartilage to bone, bilayered scaffolds consisting of PLGA, PLGA-grafted nono-hydroxyapatite (nHA-g-PLGA), and CS have been employed (**Figure 8B**). Polylactic acid (PLA) rods 200-300 µm in length served as porogens in molds filled with the PLGA/CS hydrogel. Lyophilized nHA-g-PLGA/CS constructs were placed in bone. The seeded ASCs aggregated near cartilage, and the PLA rods were removed. The chondrogenic cell spheroids were distributed throughout the bony scaffold and played roles in both cartilage and bone regeneration 112.

#### Liver

**Figure 9:**
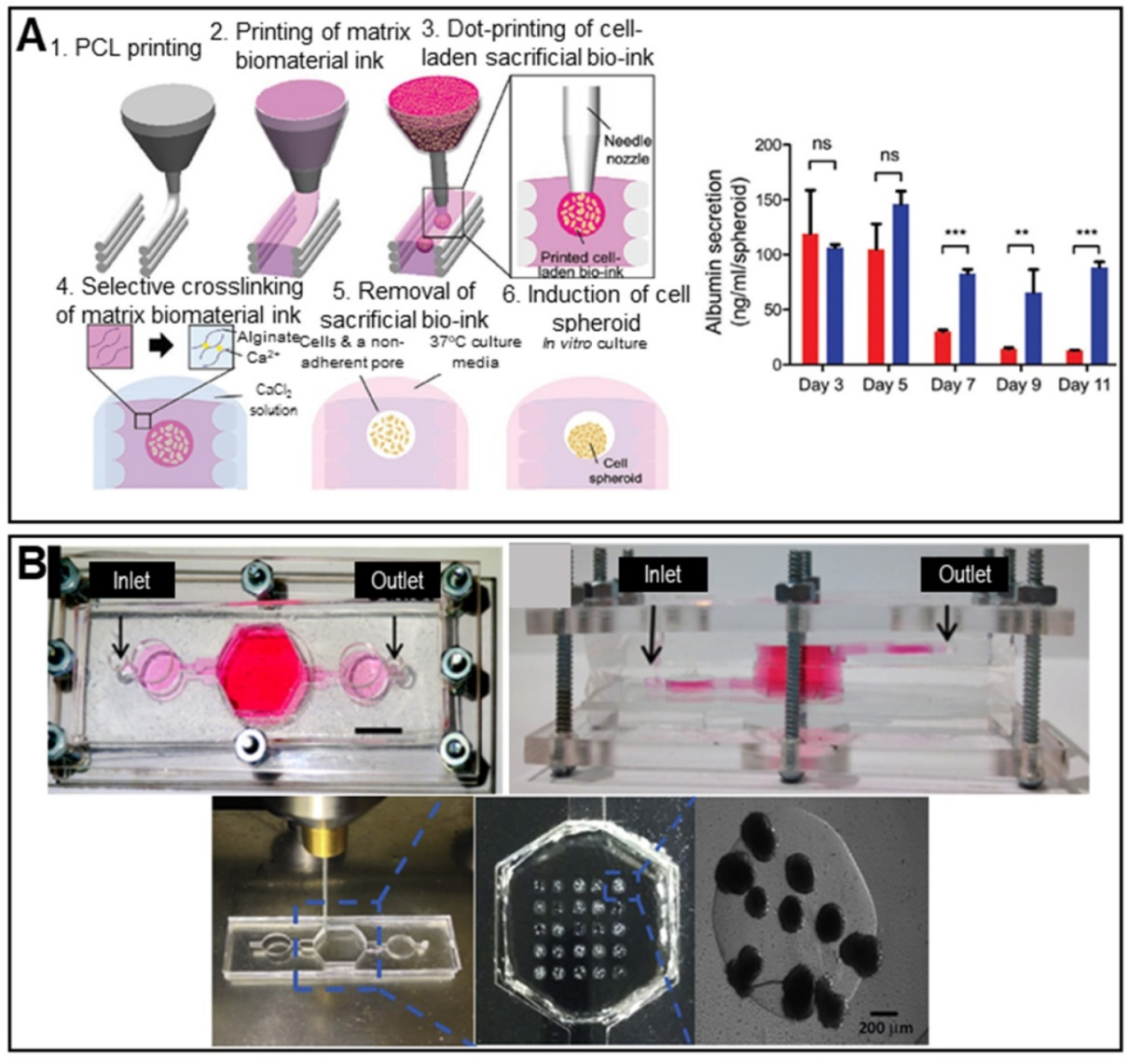
** Scaffolded spheroid applications for liver tissue regeneration. A.** Schematic illustration of *in situ* formation of spheroids (left) and a graph showing albumin secretion by conventional (red color bar) and biodot-printed phenyl-methylene hydantoin spheroids (blue color bar) (right). Adapted with permission from 54, copyright 2020 John Wiley and Sons. **B.** Macroscopic image of the bioreactor culture platform (top) and images of spheroids printing process (bottom). Adapted with permission from 114, copyright 2020 John Wiley and Sons.

Decellularized tissues are becoming the gold standard for liver TE given the biochemical similarities to native tissues. Decellularized liver tissues are routinely used to induce maturation of hepatocyte-like cells from mouse BM-MSCs 113. Spheroids cultured with decellularized liver scaffolds exhibited significantly enhanced albumin and urea secretion. The principal drawback of conventional spheroid fabrication is the inability to precisely control the spheroid diameter. The 3D bio-dot printing method, in which cell-laden spheroids are ejected directly into a sacrificial bio-ink (an alginate hydrogel) and then into a pre-printed polycaprolactone (PCL) mold, shows promise in terms of diameter control (**Figure 9A**) 54. The novel spheroids exhibited significantly higher albumin secretion compared to conventional spheroids.

As results from animal models may not adequately represent drug toxicity in humans, liver-on-a-chip models are regularly used to this end. The incorporation of spheroids into liver-on-a-chip models creates an *in vivo-*like environment, improving model reliability 114. HepG2/C3A spheroids encapsulated in 3D hepatic constructs have been used to study acetaminophen toxicity (**Figure 9B**). A 3D hepatic construct was fabricated using a 3D bioprinter and photo-crosslinkable gelatin methacrylate (GelMA) and HepG2/C3A spheroids. The printed structure was packaged in a PDMS/poly(methyl methacrylate) (PMMA) bioreactor.

The recent introduction of 3D spheroid bioprinting suggests that 3D-printed autologous liver grafts may find clinical applications. 3D bioprinting of toroidal, induced pluripotent stem cell (iPSC)-derived hepatic spheroids has been used to explore hepatic functions. The bioprinted, hepatic spheroid structure exhibited significantly higher expression of liver genes and proteins compared to singly dispersed hepatocytes 115.

#### Skin

**Figure 10:**
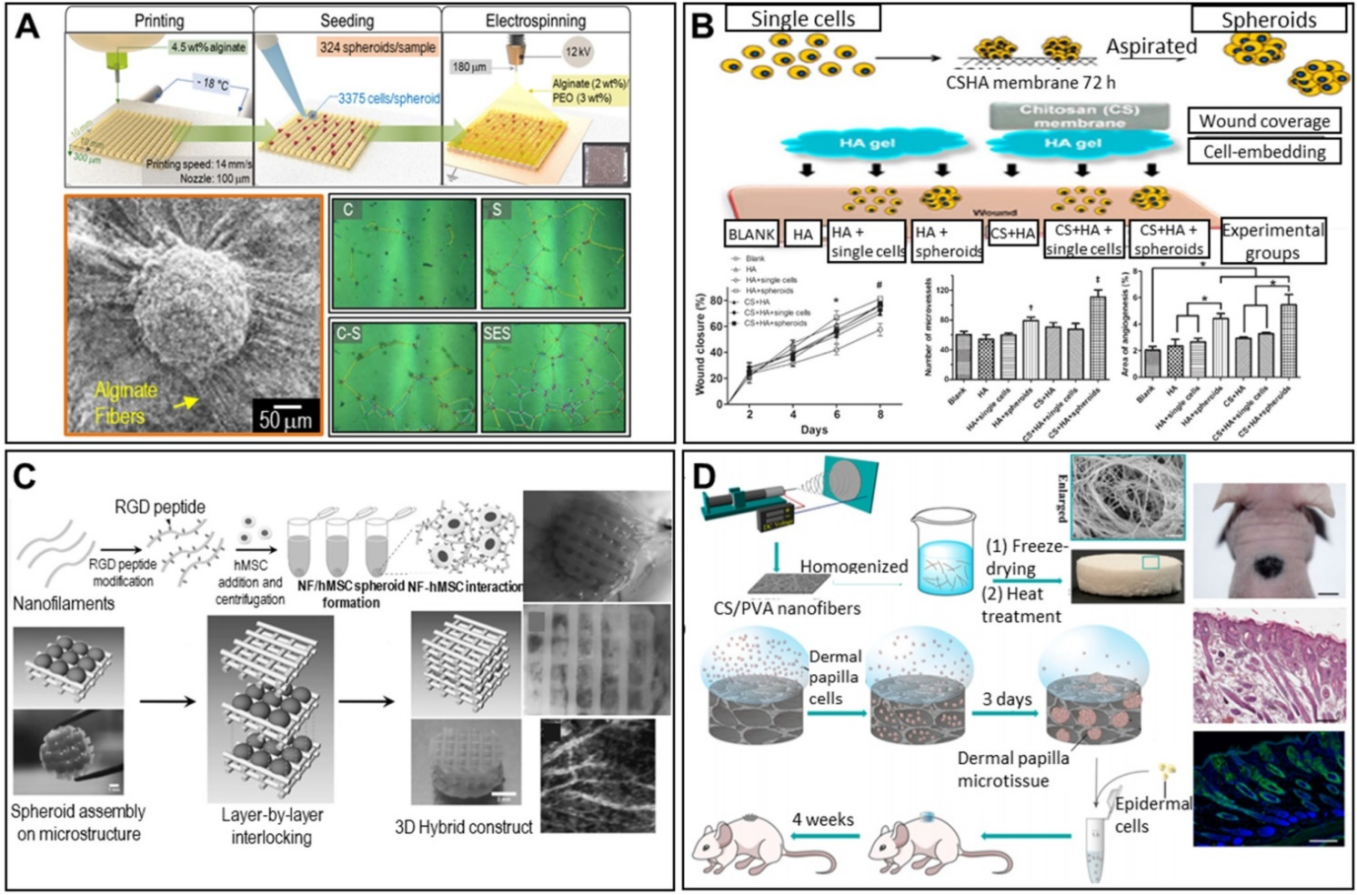
** Scaffolded spheroid applications for the regeneration of nerves, skin, vessels, and other tissues. A.** Schematic illustration of hybrid alginate scaffold fabrication to support ASC spheroids (top), with an SEM image of the fabricated scaffold (bottom left). Optical images of vessel tube formation in ASC spheroid scaffold-driven conditioned medium (right). Adapted with permission from 116, copyright 2020 IOP Publishing. **B.** Schematic diagram of the experiment (top), and wound closure/angiogenesis analysis of a rat wound model (bottom). Adapted with permission from 117, copyright 2014 John Wiley and Sons. **C.** Schematic diagram of NF/hMSC, composite, multicellular spheroid preparation (top left) and 3D biohybrid assembly of NF/hMSC composite spheroids and the scaffold microstructure (bottom left), with a macroscopic image and perilipin/PECAM-1- and bFGF-immunostained confocal microscope images of the implanted biohybrid construct (right). Adapted with permission from 119, copyright 2010 John Wiley and Sons. **D.** Schematic illustration of the cellular structure of a 3D CS/PVA NF sponge and transplantation of DP microtissue (left), with a macroscopic image (top right) (scale bar: 500 mm), H&E-stained image (middle right) (scale bar: 200 mm), and Hoechst (blue)/K14 (green) immunofluorescence image (bottom right) (scale bar: 200 mm) of 3D spheroid-treated samples. Adapted with permission from 120, copyright 2020 American Chemical Society.

Lee et al. described a spheroid-entrapping alginate hybrid obtained by seeding ASC spheroids onto a 3D-printed alginate-based mesh, followed by electrospinning of alginate/polyethylene oxide fibers directly onto the spheroids (**Figure 10A**) 116. The experimental group (with the 3D-printed/electrospun alginate scaffold and ASC spheroids) evidenced greater angiogenic gene expression (of *PECAM-1, VEGF-A,* and* HGF*) and angiogenesis-related growth factor secretion compared to two control groups (pure spheroids lacking a scaffold and single cells seeded onto an alginate scaffold), suggesting potential applications in wound healing.

Other research teams have confirmed the skin regenerative capacity of ASC spheroids in the rat skin repair model. As shown in **Figure 10B**, ASCs/spheroids were seeded into wounds in rat dorsal skin and covered with a hyaluronan gel/CS membrane to maintain a moist environment. The experiments featured a blank, the hyaluronan gel, single ASCs covered by the gel, ASC spheroids covered by the gel, the gel covered by a CS membrane, single ASCs covered by the hyaluronan gel and CS membrane, and ASC spheroids covered by the hyaluronan gel and CS membrane. Wounds treated with spheroids exhibited faster closure and significantly greater angiogenesis. Tracking of fluorescently labeled ASCs showed that the ASC spheroids were localized near micro-vessels, suggesting that angiogenesis was enhanced in a paracrine manner. Thus, based on both the *in vitro* and *in vivo* results, self-assembled ASC spheroids show promise in treatments facilitating skin TE and wound regeneration 117.

#### Vessels

To mimic the layered structure of the native vasculature, Shimazu et al. developed a double-layered vascular structure with a HUVEC core and a shell composed of human aortic SMCs 118. A gold needle coated with HUVECs was placed in the center of the chamber, and the rest of the chamber was filled with SMC spheroids and photo-crosslinkable GelMA. The samples were incubated for 4 days, and the gold needle was then removed, yielding a vascular-like layered structure with a HUVEC inner layer and an SMC outer layer.

The RGD-modification technique was used to mimic the ECM structure. Centrifugation of RGD-modified PLLA nanofibers (NFs) and hMSCs yielded spheroids 119. Alternate stacking of the spheroids and a pre-printed, two-layered, PCL-meshed scaffold yielded cylinders (**Figure 10C**). Given the biomimetic environment imparted by the RGD modification, enhanced neovascularization and *in vivo* vascularized adipose tissue formation were observed.

#### Other tissues

Spheroids can be used to regenerate hair follicles. An open-cell NF sponge comprising electrospun CS and polyvinyl alcohol (PVA) fibers generated dermal papilla (DP) cell spheroids 120. As shown in **Figure 10D**, DP cells were seeded into the pores of the sponge and aggregated into spheroids over 3 days. A chamber patch assay of a combination of DP spheroids and epidermal cells revealed significantly elevated hair follicle formation, as revealed by hematoxylin-and-eosin (H&E) staining. Additionally, immunofluorescent staining for keratin (a common hair protein) was positive, indicating successful regeneration of hair follicles.

## Conclusion

Spheroids engage in intense cell-cell and cell-matrix interactions, which elevate growth factor secretion, thus improving cellular activity, organ-specific gene expression, and cytokine production compared to 2D monocultured cells. Spheroids are robust to external stimuli, including drug entrance. As such, spheroids are attractive building blocks for TE. We reviewed some key examples of spheroid applications in TE and bioreactors, dividing the applications into approaches based on “*scaffold-free spheroids*” and “*scaffolded spheroids.*” Current spheroid applications seek to enhance tissue regeneration and 3D structure formation using Kenzan needles or 3D printers. However, spheroids do not adequately mimic multiplex cellular tissues that make up native organs. It is essential to identify the specific biochemical/mechanical factors responsible for spheroid formation. Additionally, the large diffusion gradients and low cell proliferation of spheroids must be improved. Despite these issues, spheroids are projected to play critical roles in tissue regeneration. We strongly believe that future developments will facilitate the personalized treatment of various diseases and improve disease prognoses.

## Figures and Tables

**Table 1:** Scaffold-free spheroid applications according to fabrication method

Fabrication method	Target organs/tissues	Cell types	Materials	Methods	Objectives	References
Self-assembly	Vessel	HUVECs; human aortic SMCs; human normal dermal fibroblasts	Kenzan needle array	Kenzan method combined with 3D printing	Development of scaffold-free tubular tissues using a 3D printer for tissue remodeling and endothelialization	94
	Vessel	Human skin fibroblasts	Agarose	Seeding of spheroids onto an agarose template	Fabrication of macrovascular tubular structures consisting of multicellular spheroids	121
	-	Breast cancer cell lines (MCF-7, MDA-MB-321, and Hs-578T); Osteosarcoma cell line (SaOS-2), HUVECs	PDMS Collagen with culture media	Cells in a collagen bio-ink were seeded into a PDMS mold, which may shrink depending on the nature of the cells	Fabrication of 3D cell cultures via self-assembly of various cell types	122
	-	Mouse fibroblasts; HUVECs	PMMA	Fusion of spheroids into an organoid using a biotunable acoustic system	Development of a heterogeneous multicellular structure via biotunable, acoustic node assembly	123
	Bone/cartilage	BM-MSCs	Teflon	Loading of spheroids into a tube-shaped Teflon mold	Fabrication of constant-thickness spheroids for bone and cartilage regeneration	73
Microchannel	-	Human glioblastoma cells; HUVECs; Human lung fibroblasts	Polystyrene	Seeding of spheroids onto a tumor-derived spheroid-on-a-chip to investigate tumor angiogenesis	Development of a 3D, perfusable blood vessel network and tumor spheroids	124
	-	Human fibroblasts; human embryonic stem cells, derived human neural stem cells	Agarose	Seeding of spheroids into an agarose mold	Confirmation of the role played by the cytoskeleton in self-assembly of 3D microtissues	125
	Muscle	Mouse C2C12 myoblasts; human iPSC-derived skeletal myoblasts	PDMS Collagen	Injection of motor neuron spheroids into a chip, remote from skeletal muscle bundles	Elucidation of the pathogenesis of amyotrophic lateral sclerosis, and drug screening	126

**Table 2:** Scaffolded spheroid applications according to fabrication method

Fabrication method	Target organs/tissues	Cell types	Materials	Methods	Objectives	References
Seeding	Bone	Human ASCs	PLGA/collagen/nHAps	Seeding onto electrospun patterned NFs	Combination of robust 3D spheroid cultures with patterned micro- and nano-structures	107
	Adipose tissue	Human ASCs	PCL	Melt electrowriting	Development of sheetlike spheroid constructs that can be readily handled and transferred	25
Hydrogel contraction	Vessel	HUVECs, human SMCs	GelMA, gold needle	Contraction of spheroid-embedded hydrogels	Fabrication of perfusable and robust double-layered vascular-like structures	118
Embedding	Adipose tissue	MSCs	PLA, RGD-peptide, PCL	Stacking of spheroid and PCL layers	Development of a multiscale multifunctional assembly that mimics 3D adipose tissue	119
Printing	Liver	HepG2/C3A cells	GelMA	Printing of spheroids using a GelMA solution	Fabrication of tissue-like, spheroid-laden hydrogel constructs to create a liver-on-a- chip	127
	-	L929, Rat2, C2C12	Agarose-collagen hydrogel, PLGA	Hybrid 3D bioprinting using porous microscaffolds and extrusion-based printing	Micropipette extrusion-based bioprinting of 3D, living multicellular tissues	128

## References

[1] Moscona A, Moscona H (1952). The dissociation and aggregation of cells from organ rudiments of the early chick embryo. J Anat.

[2] Sun H, Chow EC, Liu S, Du Y, Pang KS (2008). The Caco-2 cell monolayer: usefulness and limitations. Expert Opin Drug Metab Toxicol.

[3] Batalov I, Feinberg AW (2015). Differentiation of cardiomyocytes from human pluripotent stem cells using monolayer culture: supplementary issue: stem cell biology. Biomarker insights.

[4] Cukierman E, Pankov R, Stevens DR, Yamada KM (2001). Taking cell-matrix adhesions to the third dimension. Science.

[5] Xu Z, Gao Y, Hao Y, Li E, Wang Y, Zhang J (2013). Application of a microfluidic chip-based 3D co-culture to test drug sensitivity for individualized treatment of lung cancer. Biomaterials.

[6] Flampouri E, Imar S, OConnell K, Singh B (2019). Spheroid-3D and monolayer-2D intestinal electrochemical biosensor for toxicity/viability testing: Applications in drug screening, food safety, and environmental pollutant analysis. ACS sensors.

[7] Yan X, Zhou L, Wu Z, Wang X, Chen X, Yang F (2019). High throughput scaffold-based 3D micro-tumor array for efficient drug screening and chemosensitivity testing. Biomaterials.

[8] Edmondson R, Broglie JJ, Adcock AF, Yang L (2014). Three-dimensional cell culture systems and their applications in drug discovery and cell-based biosensors. Assay Drug Dev Technol.

[9] Rosso F, Giordano A, Barbarisi M, Barbarisi A (2004). From cell-ECM interactions to tissue engineering. J Cell Physiol.

[10] Wan AM, Inal S, Williams T, Wang K, Leleux P, Estevez L (2015). 3D Conducting Polymer Platforms for Electrical Control of Protein Conformation and Cellular Functions. J Mater Chem B.

[11] Bonnier F, Keating ME, Wrobel TP, Majzner K, Baranska M, Garcia-Munoz A (2015). Cell viability assessment using the Alamar blue assay: a comparison of 2D and 3D cell culture models. Toxicol *In vitro*.

[12] Chitcholtan K, Asselin E, Parent S, Sykes PH, Evans JJ (2013). Differences in growth properties of endometrial cancer in three dimensional (3D) culture and 2D cell monolayer. Exp Cell Research.

[13] Sakai Y, Yamagami S, Nakazawa K (2010). Comparative analysis of gene expression in rat liver tissue and monolayer-and spheroid-cultured hepatocytes. Cells Tissues Organs.

[14] Glicklis R, Merchuk JC, Cohen S (2004). Modeling mass transfer in hepatocyte spheroids via cell viability, spheroid size, and hepatocellular functions. Biotechnol Bioeng.

[15] Sakai Y, Nakazawa K (2007). Technique for the control of spheroid diameter using microfabricated chips. Acta biomater.

[16] Groebe K, Mueller-Klieser W (1996). On the relation between size of necrosis and diameter of tumor spheroids. Int J Radiat Oncol Biol Phys.

[17] Mihara H, Kugawa M, Sayo K, Tao F, Shinohara M, Nishikawa M (2019). Improved oxygen supply to multicellular spheroids using a gas-permeable plate and embedded hydrogel beads. Cells.

[18] Barisam M, Saidi MS, Kashaninejad N, Nguyen N-T (2018). Prediction of necrotic core and hypoxic zone of multicellular spheroids in a microbioreactor with a u-shaped barrier. Micromachines.

[19] Ahmed HMM, Salerno S, Piscioneri A, Khakpour S, Giorno L, De Bartolo L (2017). Human liver microtissue spheroids in hollow fiber membrane bioreactor. Colloids Surf B.

[20] Wei J, Lei D, Chen M, Ran P, Li X (2020). Engineering HepG2 spheroids with injectable fiber fragments as predictable models for drug metabolism and tumor infiltration. J Biomed Mater Res Part B Appl Biomater.

[21] Costa EC, Silva DN, Moreira AF, Correia IJ (2019). Optical clearing methods: An overview of the techniques used for the imaging of 3D spheroids. Biotechnol Bioeng.

[22] Xie P, Zhao C, Liang X, Huang W, Chen Y, Cai Z (2020). Preparation of frozen sections of multicellular tumor spheroids coated with ice for mass spectrometry imaging. Anal Chem.

[23] Dutta RC, Dutta AK (2010). Comprehension of ECM-Cell dynamics: A prerequisite for tissue regeneration. Biotechnology Adv.

[24] Lee HW, Kook Y-M, Lee HJ, Park H, Koh W-G (2014). A three-dimensional co-culture of HepG2 spheroids and fibroblasts using double-layered fibrous scaffolds incorporated with hydrogel micropatterns. RSC Adv.

[25] McMaster R, Hoefner C, Hrynevich A, Blum C, Wiesner M, Wittmann K (2019). Tailored Melt Electrowritten Scaffolds for the Generation of Sheet-Like Tissue Constructs from Multicellular Spheroids. Adv Healthc Mater.

[26] Loessner D, Stok KS, Lutolf MP, Hutmacher DW, Clements JA, Rizzi SC (2010). Bioengineered 3D platform to explore cell-ECM interactions and drug resistance of epithelial ovarian cancer cells. Biomaterials.

[27] Badylak SF, Gilbert TW (2008). Immune response to biologic scaffold materials. Semin Immunol.: Elsevier.

[28] Lin RZ, Chou LF, Chien CC, Chang HY (2006). Dynamic analysis of hepatoma spheroid formation: roles of E-cadherin and beta1-integrin. Cell Tissue Res.

[29] Robinson EE, Zazzali KM, Corbett SA, Foty RA (2003). Alpha5beta1 integrin mediates strong tissue cohesion. J Cell Sci.

[30] Takeichi M (1988). The cadherins: cell-cell adhesion molecules controlling animal morphogenesis. Development.

[31] Liu FF, Peng C, Escher BI, Fantino E, Giles C, Were S (2013). Hanging drop: an *in vitro* air toxic exposure model using human lung cells in 2D and 3D structures. J Hazard Mater.

[32] Kelm JM, Timmins NE, Brown CJ, Fussenegger M, Nielsen LK (2003). Method for generation of homogeneous multicellular tumor spheroids applicable to a wide variety of cell types. Biotechnol Bioeng.

[33] Koide N, Sakaguchi K, Koide Y, Asano K, Kawaguchi M, Matsushima H (1990). Formation of multicellular spheroids composed of adult rat hepatocytes in dishes with positively charged surfaces and under other nonadherent environments. Exp Cell Res.

[34] Zhang S, Liu P, Chen L, Wang Y, Wang Z, Zhang B (2015). The effects of spheroid formation of adipose-derived stem cells in a microgravity bioreactor on stemness properties and therapeutic potential. Biomaterials.

[35] Nyberg SL, Hardin J, Amiot B, Argikar UA, Remmel RP, Rinaldo P (2005). Rapid, large-scale formation of porcine hepatocyte spheroids in a novel spheroid reservoir bioartificial liver. Liver Transpl.

[36] Hammond T, Hammond J (2001). Optimized suspension culture: the rotating-wall vessel. Am J Physiol Renal Physiol.

[37] Carpenedo RL, Sargent CY, McDevitt TC (2007). Rotary suspension culture enhances the efficiency, yield, and homogeneity of embryoid body differentiation. Stem Cells.

[38] Shin CS, Kwak B, Han B, Park K (2013). Development of an *in vitro* 3D tumor model to study therapeutic efficiency of an anticancer drug. Mol Pharm.

[39] Tung Y-C, Hsiao AY, Allen SG, Torisawa Y-s, Ho M, Takayama S (2011). High-throughput 3D spheroid culture and drug testing using a 384 hanging drop array. Analyst.

[40] Jeong SY, Lee JH, Shin Y, Chung S, Kuh HJ (2016). Co-Culture of Tumor Spheroids and Fibroblasts in a Collagen Matrix-Incorporated Microfluidic Chip Mimics Reciprocal Activation in Solid Tumor Microenvironment. PLoS One.

[41] Yoon S, Kim JA, Lee SH, Kim M, Park TH (2013). Droplet-based microfluidic system to form and separate multicellular spheroids using magnetic nanoparticles. Lab on a Chip.

[42] Lee D, Cha C (2018). The combined effects of co-culture and substrate mechanics on 3D tumor spheroid formation within microgels prepared via flow-focusing microfluidic fabrication. Pharmaceutics.

[43] Wang Y, Wang J (2014). Mixed hydrogel bead-based tumor spheroid formation and anticancer drug testing. Analyst.

[44] Chen Z, Wang L, Stegemann JP (2011). Phase-separated chitosan-fibrin microbeads for cell delivery. J Microencapsul.

[45] Napolitano AP, Chai P, Dean DM, Morgan JR (2007). Dynamics of the self-assembly of complex cellular aggregates on micromolded nonadhesive hydrogels. Tissue Eng.

[46] Fukuda J, Khademhosseini A, Yeo Y, Yang X, Yeh J, Eng G (2006). Micromolding of photocrosslinkable chitosan hydrogel for spheroid microarray and co-cultures. Biomaterials.

[47] Silva KR, Rezende RA, Pereira FD, Gruber P, Stuart MP, Ovsianikov A (2016). Delivery of human adipose stem cells spheroids into lockyballs. PloS one.

[48] Chen K, Wu M, Guo F, Li P, Chan CY, Mao Z (2016). Rapid formation of size-controllable multicellular spheroids via 3D acoustic tweezers. Lab Chip.

[49] Chen B, Wu Y, Ao Z, Cai H, Nunez A, Liu Y (2019). High-throughput acoustofluidic fabrication of tumor spheroids. Lab Chip.

[50] Tait A, Glynne-Jones P, Hill AR, Smart DE, Blume C, Hammarstrom B (2019). Engineering multi-layered tissue constructs using acoustic levitation. Sci Rep.

[51] Anupam A (2017). High throughput fabrication of cell spheroids by templating water-in-water Pickering emulsions. Mater Horiz.

[52] Wang A, Madden LA, Paunov VN (2020). Advanced biomedical applications based on emerging 3D cell culturing platforms. J Mater Chem B.

[53] Utama RH, Atapattu L, O'Mahony AP, Fife CM, Baek J, Allard T (2020). A 3D bioprinter specifically designed for the high-throughput production of matrix-embedded multicellular spheroids. Iscience.

[54] Jeon S, Heo JH, Kim MK, Jeong W, Kang HW High-Precision 3D Bio-Dot Printing to Improve Paracrine Interaction between Multiple Types of Cell Spheroids. Adv Funct Mater. 2020: 2005324.

[55] Lanza R, Langer R, Vacanti JP, Atala A. Principles of tissue engineering: Academic press; 2020

[56] Ovsianikov A, Khademhosseini A, Mironov V (2018). The Synergy of Scaffold-Based and Scaffold-Free Tissue Engineering Strategies. Trends Biotechnol.

[57] Kim EM, Lee YB, Byun H, Chang H-k, Park J, Shin H (2018). Fabrication of Spheroids with Uniform Size by Self-Assembly of a Micro-Scaled Cell Sheet (μCS): The Effect of Cell Contraction on Spheroid Formation. ACS Appl Mater Interfaces.

[58] Zhang Q, Nguyen PD, Shi S, Burrell JC, Cullen DK, Le AD (2018). 3D bio-printed scaffold-free nerve constructs with human gingiva-derived mesenchymal stem cells promote rat facial nerve regeneration. Sci Rep.

[59] Khoshnood N, Zamaniain A A comprehensive review on scaffold-free bioinks for bioprinting. Bioprinting. 2020: e00088.

[60] Gao Q, He Y, Fu J-z, Liu A, Ma L (2015). Coaxial nozzle-assisted 3D bioprinting with built-in microchannels for nutrients delivery. Biomaterials.

[61] Dahlin C, Linde A, Gottlow J, Nyman S (1988). Healing of bone defects by guided tissue regeneration. Plast Reconstr Surg.

[62] Bose S, Roy M, Bandyopadhyay A (2012). Recent advances in bone tissue engineering scaffolds. Trends Biotechnol.

[63] Mistry AS, Mikos AG (2005). Tissue engineering strategies for bone regeneration. Adv Biochem Eng Biotechnol.

[64] Jeong J, Kim JH, Shim JH, Hwang NS, Heo CY (2019). Bioactive calcium phosphate materials and applications in bone regeneration. Biomater Res.

[65] Suenaga H, Furukawa KS, Suzuki Y, Takato T, Ushida T (2015). Bone regeneration in calvarial defects in a rat model by implantation of human bone marrow-derived mesenchymal stromal cell spheroids. J Mater Sci Mater Med.

[66] Hasani-Sadrabadi MM, Sarrion P, Pouraghaei S, Chau Y, Ansari S, Li S (2020). An engineered cell-laden adhesive hydrogel promotes craniofacial bone tissue regeneration in rats. Sci Transl Med.

[67] Filipowska J, Tomaszewski KA, Niedźwiedzki Ł, Walocha JA, Niedźwiedzki T (2017). The role of vasculature in bone development, regeneration and proper systemic functioning. Angiogenesis.

[68] Lee J, Lee S, Ahmad T, Madhurakkat Perikamana SK, Lee J, Kim EM (2020). Human adipose-derived stem cell spheroids incorporating platelet-derived growth factor (PDGF) and bio-minerals for vascularized bone tissue engineering. Biomaterials.

[69] Heo DN, Hospodiuk M, Ozbolat IT (2019). Synergistic interplay between human MSCs and HUVECs in 3D spheroids laden in collagen/fibrin hydrogels for bone tissue engineering. Acta Biomater.

[70] Kiani C, Liwen C, Wu YJ, Albert JY, Burton BY (2002). Structure and function of aggrecan. Cell research.

[71] Becerra J, Andrades JA, Guerado E, Zamora-Navas P, Lopez-Puertas JM, Reddi AH (2010). Articular cartilage: structure and regeneration. Tissue Eng Part B Rev.

[72] Fedarko NS, Vetter U, Weinstein S, Robey PG (1992). Age-related changes in hyaluronan, proteoglycan, collagen, and osteonectin synthesis by human bone cells. J Cell Physiol.

[73] Ishihara K, Nakayama K, Akieda S, Matsuda S, Iwamoto Y (2014). Simultaneous regeneration of full-thickness cartilage and subchondral bone defects *in vivo* using a three-dimensional scaffold-free autologous construct derived from high-density bone marrow-derived mesenchymal stem cells. J Orthop Surg Res.

[74] Jeon JH, Yun BG, Lim MJ, Kim SJ, Lim MH, Lim JY (2020). Rapid Cartilage Regeneration of Spheroids Composed of Human Nasal Septum-Derived Chondrocyte in Rat Osteochondral Defect Model. Tissue Eng Regen Med.

[75] Hogan BL, Barkauskas CE, Chapman HA, Epstein JA, Jain R, Hsia CC (2014). Repair and regeneration of the respiratory system: complexity, plasticity, and mechanisms of lung stem cell function. Cell stem cell.

[76] Stramiello JA, Saddawi-Konefka R, Ryan J, Brigger MT (2020). The role of 3D printing in pediatric airway obstruction: A systematic review. Int J Pediatr Otorhinolaryngol.

[77] Gao M, Zhang H, Dong W, Bai J, Gao B, Xia D (2017). Tissue-engineered trachea from a 3D-printed scaffold enhances whole-segment tracheal repair. Sci Rep.

[78] Machino R, Matsumoto K, Taniguchi D, Tsuchiya T, Takeoka Y, Taura Y (2019). Replacement of Rat Tracheas by Layered, Trachea-Like, Scaffold-Free Structures of Human Cells Using a Bio-3D Printing System. Adv Healthc Mater.

[79] Hofmann AF (1999). The continuing importance of bile acids in liver and intestinal disease. Arch Intern Med.

[80] Gaskell H, Sharma P, Colley HE, Murdoch C, Williams DP, Webb SD (2016). Characterization of a functional C3A liver spheroid model. Toxicol Res (Camb).

[81] Parola M, Pinzani M (2019). Liver fibrosis: Pathophysiology, pathogenetic targets and clinical issues. Mol Aspects Med.

[82] Mannaerts I, Eysackers N, Anne van Os E, Verhulst S, Roosens T, Smout A (2020). The fibrotic response of primary liver spheroids recapitulates *in vivo* hepatic stellate cell activation. Biomaterials.

[83] Douaud G, Groves AR, Tamnes CK, Westlye LT, Duff EP, Engvig A (2014). A common brain network links development, aging, and vulnerability to disease. PNAS.

[84] Lou Y, Huang P, Li D, Cen Z, Wang B, Gao J (2015). Altered brain network centrality in depressed Parkinson's disease patients. J Mov Disord.

[85] Takeuchi H, Ikeguchi R, Aoyama T, Oda H, Yurie H, Mitsuzawa S (2020). A scaffold-free Bio 3D nerve conduit for repair of a 10-mm peripheral nerve defect in the rats. Microsurgery.

[86] Martin P (1997). Wound healing - aiming for perfect skin regeneration. Science.

[87] Barrientos S, Stojadinovic O, Golinko MS, Brem H, Tomic-Canic M (2008). Growth factors and cytokines in wound healing. Wound Repair Regen.

[88] Santos JM, Camoes SP, Filipe E, Cipriano M, Barcia RN, Filipe M (2015). Three-dimensional spheroid cell culture of umbilical cord tissue-derived mesenchymal stromal cells leads to enhanced paracrine induction of wound healing. Stem Cell Res Ther.

[89] Park I-S, Chung P-S, Ahn JC (2015). Enhancement of ischemic wound healing by spheroid grafting of human adipose-derived stem cells treated with low-level light irradiation. PLoS One.

[90] Mvula B, Mathope T, Moore T, Abrahamse H (2008). The effect of low level laser irradiation on adult human adipose derived stem cells. Lasers Med Science.

[91] Carmeliet P, Jain RK (2011). Molecular mechanisms and clinical applications of angiogenesis. Nature.

[92] Potente M, Gerhardt H, Carmeliet P (2011). Basic and therapeutic aspects of angiogenesis. Cell.

[93] Lee JH, Han YS, Lee SH (2016). Long-Duration Three-Dimensional Spheroid Culture Promotes Angiogenic Activities of Adipose-Derived Mesenchymal Stem Cells. Biomol Ther (Seoul).

[94] Itoh M, Nakayama K, Noguchi R, Kamohara K, Furukawa K, Uchihashi K (2015). Scaffold-Free Tubular Tissues Created by a Bio-3D Printer Undergo Remodeling and Endothelialization when Implanted in Rat Aortae. PLoS One.

[95] Richards DJ, Tan Y, Jia J, Yao H, Mei Y (2013). 3D printing for tissue engineering. Isr J Chem.

[96] Noguchi R, Nakayama K, Itoh M, Kamohara K, Furukawa K, Oyama JI (2016). Development of a three-dimensional pre-vascularized scaffold-free contractile cardiac patch for treating heart disease. J Heart Lung Transplant.

[97] Daly AC, Davidson MD, Burdick JA (2021). 3D bioprinting of high cell-density heterogeneous tissue models through spheroid fusion within self-healing hydrogels. Nat commun.

[98] Shen D, Cheng K, Marbán E (2012). Dose-dependent functional benefit of human cardiosphere transplantation in mice with acute myocardial infarction. J Cell Mol Med.

[99] Park YS, Hwang J-Y, Jun Y, Jin YM, Kim G, Kim HY (2016). Scaffold-free parathyroid tissue engineering using tonsil-derived mesenchymal stem cells. Acta Biomater.

[100] Lee JS-j, Kim S-j, Choi JS, Eom MR, Shin H, Kwon SK (2020). Adipose-derived mesenchymal stem cell spheroid sheet accelerates regeneration of ulcerated oral mucosa by enhancing inherent therapeutic properties. J Ind Eng Chem.

[101] Pihlstrom BL, Michalowicz BS, Johnson NW (2005). Periodontal diseases. The lancet.

[102] Sano K, Usui M, Moritani Y, Nakazawa K, Hanatani T, Kondo H (2020). Co-cultured spheroids of human periodontal ligament mesenchymal stem cells and vascular endothelial cells enhance periodontal tissue regeneration. Regen Ther.

[103] Henry E, Cores J, Hensley MT, Anthony S, Vandergriff A, de Andrade JB (2015). Adult lung spheroid cells contain progenitor cells and mediate regeneration in rodents with bleomycin-induced pulmonary fibrosis. Stem Cells Transl Med.

[104] Dinh P-UC, Paudel D, Brochu H, Popowski KD, Gracieux MC, Cores J (2020). Inhalation of lung spheroid cell secretome and exosomes promotes lung repair in pulmonary fibrosis. Nat Commun.

[105] Kim HJ, Kim U-J, Vunjak-Novakovic G, Min B-H, Kaplan DL (2005). Influence of macroporous protein scaffolds on bone tissue engineering from bone marrow stem cells. Biomaterials.

[106] Maji S, Agarwal T, Das J, Maiti TK (2018). Development of gelatin/carboxymethyl chitosan/nano-hydroxyapatite composite 3D macroporous scaffold for bone tissue engineering applications. Carbohydr Polym.

[107] Sankar S, Sharma CS, Rath SN (2019). Enhanced osteodifferentiation of MSC spheroids on patterned electrospun fiber mats - An advanced 3D double strategy for bone tissue regeneration. Mater Sci Eng C Mater Biol Appl.

[108] Favreau H, Pijnenburg L, Seitlinger J, Fioretti F, Keller L, Scipioni D (2020). Osteochondral repair combining therapeutics implant with mesenchymal stem cells spheroids. Nanomedicine: NBM.

[109] O'Shea TM, Miao X (2008). Bilayered scaffolds for osteochondral tissue engineering. Tissue Eng. Part B Rev.

[110] Huang G, Tseng C, Dai L, Hsieh P, Hsu S (2013). Solid freeform-fabricated scaffolds designed to carry multicellular mesenchymal stem cell spheroids for cartilage regeneration. Eur Cells Mater.

[111] Zhang K, Yan S, Li G, Cui L, Yin J (2015). In-situ birth of MSCs multicellular spheroids in poly (L-glutamic acid)/chitosan scaffold for hyaline-like cartilage regeneration. Biomaterials.

[112] Qin Y, Li G, Wang C, Zhang D, Zhang L, Fang H (2020). Biomimetic Bilayer Scaffold as an Incubator to Induce Sequential Chondrogenesis and Osteogenesis of Adipose Derived Stem Cells for Construction of Osteochondral Tissue. ACS Biomater Sci Eng.

[113] Wu Q, Tang J, Li Y, Li L, Wang Y, Bao J (2017). Hepatic differentiation of mouse bone marrow-derived mesenchymal stem cells using a novel 3D culture system. Mol Med Rep.

[114] Nie J, Gao Q, Fu J, He Y (2020). Grafting of 3D bioprinting to *in vitro* drug screening: a review. Adv Healthc Mater.

[115] Goulart E, de Caires-Junior LC, Telles-Silva KA, Araujo BHS, Rocco SA, Sforca M (2019). 3D bioprinting of liver spheroids derived from human induced pluripotent stem cells sustain liver function and viability *in vitro*. Biofabrication.

[116] Lee JS, Chae S, Yoon D, Yoon D, Chun W, Kim GH (2020). Angiogenic factors secreted from human ASC spheroids entrapped in an alginate-based hierarchical structure via combined 3D printing/electrospinning system. Biofabrication.

[117] Hsu SH, Hsieh PS (2015). Self-assembled adult adipose-derived stem cell spheroids combined with biomaterials promote wound healing in a rat skin repair model. Wound Repair Regen.

[118] Shimazu Y, Zhang B, Yue Z, Wallace GG, Fukuda J (2019). Engineering of perfusable double-layered vascular structures using contraction of spheroid-embedded hydrogel and electrochemical cell detachment. J Biosci Bioeng.

[119] Kim TG, Park SH, Chung HJ, Yang DY, Park TG (2010). Hierarchically assembled mesenchymal stem cell spheroids using biomimicking nanofilaments and microstructured scaffolds for vascularized adipose tissue engineering. Adv Funct Mater.

[120] Zhang K, Bai X, Yuan Z, Cao X, Jiao X, Qin Y (2020). Cellular Nanofiber Structure with Secretory Activity-Promoting Characteristics for Multicellular Spheroid Formation and Hair Follicle Regeneration. ACS Appl Mater Interfaces.

[121] Norotte C, Marga FS, Niklason LE, Forgacs G (2009). Scaffold-free vascular tissue engineering using bioprinting. Biomaterials.

[122] Shahin-Shamsabadi A, Selvaganapathy PR (2019). A rapid biofabrication technique for self-assembled collagen-based multicellular and heterogeneous 3D tissue constructs. Acta Biomater.

[123] Chen P, Güven S, Usta OB, Yarmush ML, Demirci U (2015). Biotunable acoustic node assembly of organoids. Adv Healthc Mater.

[124] Ko J, Ahn J, Kim S, Lee Y, Lee J, Park D (2019). Tumor spheroid-on-a-chip: a standardized microfluidic culture platform for investigating tumor angiogenesis. Lab on a Chip.

[125] Dean DM, Rago AP, Morgan JR (2009). Fibroblast elongation and dendritic extensions in constrained versus unconstrained microtissues. Cell Motil Cytoskeleton.

[126] Osaki T, Uzel SG, Kamm RD (2018). Microphysiological 3D model of amyotrophic lateral sclerosis (ALS) from human iPS-derived muscle cells and optogenetic motor neurons. Sci Adv.

[127] Bhise NS, Manoharan V, Massa S, Tamayol A, Ghaderi M, Miscuglio M (2016). A liver-on-a-chip platform with bioprinted hepatic spheroids. Biofabrication.

[128] Tan YJ, Tan X, Yeong WY, Tor SB (2016). Hybrid microscaffold-based 3D bioprinting of multi-cellular constructs with high compressive strength: a new biofabrication strategy. Sci Rep.

